# In-hospital arrhythmic burden reduction in diabetic patients with acute myocardial infarction treated with SGLT2-inhibitors: Insights from the SGLT2-I AMI PROTECT study

**DOI:** 10.3389/fcvm.2022.1012220

**Published:** 2022-09-27

**Authors:** Arturo Cesaro, Felice Gragnano, Pasquale Paolisso, Luca Bergamaschi, Emanuele Gallinoro, Celestino Sardu, Niya Mileva, Alberto Foà, Matteo Armillotta, Angelo Sansonetti, Sara Amicone, Andrea Impellizzeri, Giuseppe Esposito, Nuccia Morici, Jacopo Andrea Oreglia, Gianni Casella, Ciro Mauro, Dobrin Vassilev, Nazzareno Galie, Gaetano Santulli, Carmine Pizzi, Emanuele Barbato, Paolo Calabrò, Raffaele Marfella

**Affiliations:** ^1^Department of Translational Medical Sciences, University of Campania ‘Luigi Vanvitelli', Naples, Italy; ^2^Division of Cardiology, A.O.R.N. “Sant'Anna e San Sebastiano”, Caserta, Italy; ^3^Cardiovascular Center Aalst, OLV-Clinic, Aalst, Belgium; ^4^Department of Advanced Biomedical Sciences, University Federico II, Naples, Italy; ^5^Cardiology Unit, IRCCS Azienda Ospedaliero-Universitaria di Bologna, Bologna, Italy; ^6^Department of Experimental, Diagnostic and Specialty Medicine-DIMES, University of Bologna, Bologna, Italy; ^7^Department of Advanced Medical and Surgical Sciences, University of Campania “Luigi Vanvitelli”, Naples, Italy; ^8^Cardiology Clinic, “Alexandrovska” University Hospital, Medical University of Sofia, Sofia, Bulgaria; ^9^Interventional Cardiology Unit, De Gasperis Cardio Center, Niguarda Hospital, Milan, Italy; ^10^IRCCS S. Maria Nascente - Fondazione Don Carlo Gnocchi ONLUS, Milan, Italy; ^11^Unit of Cardiology, Maggiore Hospital, Bologna, Italy; ^12^Department of Cardiology, Hospital Cardarelli, Naples, Italy; ^13^Medica Cor Hospital, Ruse, Bulgaria; ^14^International Translational Research and Medical Education (ITME) Consortium, Naples, Italy; ^15^Department of Medicine (Division of Cardiology) and Department of Molecular Pharmacology, Wilf Family Cardiovascular Research Institute, Einstein-Sinai Diabetes Research Center, The Fleischer Institute for Diabetes and Metabolism, Albert Einstein College of Medicine, New York, NY, United States; ^16^Mediterranea Cardiocentro, Naples, Italy

**Keywords:** sodium-glucose cotransporter 2 inhibitors (SGLT2-i), acute myocardial infarction, atrial fibrillation, ventricular arrhythmias, ventricular tachycardia, hyperglycemia

## Abstract

**Background:**

Sodium-glucose co-transporter 2 inhibitors (SGLT2-i) have shown significant cardiovascular benefits in patients with and without type 2 diabetes mellitus (T2DM). They have also gained interest for their potential anti-arrhythmic role and their ability to reduce the occurrence of atrial fibrillation (AF) and ventricular arrhythmias (VAs) in T2DM and heart failure patients.

**Objectives:**

To investigate in-hospital new-onset cardiac arrhythmias in a cohort of T2DM patients presenting with acute myocardial infarction (AMI) treated with SGLT2-i vs. other oral anti-diabetic agents (non-SGLT2-i users).

**Methods:**

Patients from the SGLT2-I AMI PROTECT registry (NCT05261867) were stratified according to the use of SGLT2-i before admission for AMI, divided into SGLT2-i users vs. non-SGLT2-i users. In-hospital outcomes included the occurrence of in-hospital new-onset cardiac arrhythmias (NOCAs), defined as a composite of new-onset AF and sustained new-onset ventricular tachycardia (VT) and/or ventricular fibrillation (VF) during hospitalization.

**Results:**

The study population comprised 646 AMI patients categorized into SGLT2-i users (111 patients) and non-SGLT2-i users (535 patients). SGLT2-i users had a lower rate of NOCAs compared with non-SGLT2-i users (6.3 vs. 15.7%, *p* = 0.010). Moreover, SGLT2-i was associated with a lower rate of AF and VT/VF considered individually (*p* = 0.032). In the multivariate logistic regression model, after adjusting for all confounding factors, the use of SGLT2-i was identified as an independent predictor of the lower occurrence of NOCAs (OR = 0.35; 95%CI 0.14–0.86; *p* = 0.022). At multinomial logistic regression, after adjusting for potential confounders, SGLT2-i therapy remained an independent predictor of VT/VF occurrence (OR = 0.20; 95%CI 0.04–0.97; *p* = 0.046) but not of AF occurrence.

**Conclusions:**

In T2DM patients, the use of SGLT2-i was associated with a lower risk of new-onset arrhythmic events during hospitalization for AMI. In particular, the primary effect was expressed in the reduction of VAs. These findings emphasize the cardioprotective effects of SGLT2-i in the setting of AMI beyond glycemic control.

**Trial registration:**

Data are part of the observational international registry: SGLT2-I AMI PROTECT. ClinicalTrials.gov, identifier: NCT05261867.

## Introduction

Cardiac arrhythmias, specifically atrial fibrillation (AF) and ventricular arrhythmias (VAs), are common in the early phase of acute myocardial infarction (AMI), in both ST-segment elevation (STEMI) and non-ST-segment elevation myocardial infarction (NSTEMI) (1–3). Ischemia in myocardial infarction induces heterogeneity in excitability, refractoriness, and conduction, thus creating the substrate for the onset of potentially life-threatening arrhythmias (4–6). AF as new-onset arrhythmia occurs in about 5% of cases after STEMI, and the risk of new-onset AF in AMI patients increases by 60–77% (1, 2, 7). VAs, including ventricular tachycardia (VT) and/or ventricular fibrillation (VF), may occur at any time during AMI, especially in the inpatient phase, and represent a leading complication in this setting (1, 8). Although the incidence of VAs has declined in the hospital phase, mainly due to prompt revascularization with primary percutaneous coronary intervention (pPCI) and the early administration of medical therapy, the risk of arrhythmias, cardiac arrest, and sudden cardiac death remains high (1, 9–11). Moreover, the occurrence of VAs in early phase of AMI has been associated with an increased death rate during in-hospital period and 6-month follow-up (5, 12).

Sodium-glucose cotransporter 2 inhibitors (SGLT2-i) are a new class of drugs that act by inhibiting the reabsorption of sodium and glucose in the proximal tubules of the kidney. They were introduced as oral anti-diabetic drugs in patients with type 2 diabetes mellitus (T2DM), and later showed strong evidence of cardiovascular benefits in diabetic and non-diabetic patients with atherosclerotic cardiovascular disease and heart failure (HF), irrespective of the ejection fraction (13–16). In T2DM patients, the SGLT2-i have been shown to improve cardiac function (17), reduce hospitalizations, and positively impact prognosis in patients with stable ischemic heart disease undergoing surgical revascularization (18). In addition, they have an ameliorative effect on sympathetic tone, potentially reducing arrhythmic burden (19). Patients with AMI, even those with T2DM, are prone to developing new-onset arrhythmias due to autonomic, electrical, and structural remodeling, and glycemic fluctuations (20, 21). Although there is evidence for a reduction in new-onset arrhythmic events in patients treated with SGLT2-i, previous studies yielded inconclusive results and a variable association between SGLT2-i treatment and cardiac arrhythmias (22–24). Furthermore, the efficacy of SGLT2-i in preventing arrhythmic events in specific settings of diabetic patients with AMI remains unclear. To address this uncertainty, we evaluated the effect of SGLT2-i on the occurrence of new arrhythmic events during hospitalization in diabetic patients presenting with AMI.

## Methods

### Study design and population

The Cardioprotective Effect of Sodium-Glucose Cotransporter-2 Inhibitors in Diabetic Patients With Acute Myocardial Infarction registry (SGLT2-I AMI PROTECT, ClinicalTrials.gov, identifier: NCT05261867) is a multicenter international observational registry that collects data on consecutive diabetic patients admitted with AMI, both NSTEMI and STEMI, undergoing percutaneous coronary intervention (PCI), between January 2018 and November 2021. The study design and main results have been previously reported (25). Based on admission drug therapy, patients were divided into two groups: *SGLT2-i users*, if they were admitted to chronic SGLT2-i therapy (defined as ongoing treatment for at least 3 months before hospitalization) and *non-SGLT2-i users*, if they received other oral anti-diabetic (OAD) treatment. Patients on insulin therapy before hospitalization or with incomplete information on medical therapy at admission were excluded. Patients who started SGLT2-i therapy after the acute index event, or patients with type I diabetes mellitus, treated with coronary artery bypass grafting (CABG) as a revascularization strategy, with severe valvular heart disease, severe anemia, a history of bleeding, pulmonary embolism, glomerular filtration rate < 30 ml/min/1.73 m^2^ and malignancies were excluded. Patients with more than 20% missing values in the collected data were also excluded due to potential bias. All patients were treated with optimal medical therapy, in accordance with current guidelines. The current study was performed in accordance with the guidelines of the Declaration of Helsinki, and all patients provided informed consent.

### Study endpoints

The primary endpoint was the occurrence of in-hospital new-onset cardiac arrhythmias (NOCAs), defined as a composite of new-onset AF and new-onset sustained VT/VF during hospitalization. New-onset of AF or sustained VT/VF was considered any detection of AF or VT/VF, as defined by international guidelines, using telemetry recording or electrocardiogram throughout hospitalization from admission to discharge in patients with no previous history of AF or established tachyarrhythmia disorders. An analysis of the individual components (AF and VT/VF) of the composite endpoint was performed.

### Statistical analysis

Continuous variables are presented as mean and standard deviation (SD) or median and interquartile range, and categorical variables as numbers and percentages. The normal distribution was first assessed using the Kolmogorov–Smirnov goodness-of-fit test. Categorical data were compared using either the Pearson chi-square test or the Fisher exact test when appropriate, and continuous variables were compared using the non-parametric Mann–Whitney *U*-test, Student's *t*-test, or a one-way ANOVA followed by Tukey-Kramer post hoc correction, as appropriate. Univariate analysis was performed to identify variables associated with arrhythmic events. All variables found to have a *P*-value ≤0.1 in the univariate evaluation were considered to be candidates for a subsequent multivariate logistic regression analysis. We performed multivariate hierarchical binary logistic regression and multinomial logistic regression models to assess the predictability of variables on the occurrence of composite endpoints, and each of the components is presented as odds ratios (ORs) and 95% confidence intervals (CIs). Statistical significance was set as *P* < 0.05. All analyses were performed with the Statistical Package for the Social Sciences software version 25 (SPSS, IBM^®^, Armonk, New York) and R software (CRAN^®^ 3.3.4).

## Results

### Study population and baseline characteristics

In the SGLT2-i AMI PROTECT registry, 1,118 diabetic patients with AMI were screened. After excluding non-eligible patients 646 patients were considered for the present analysis. According to the use of SGLT2-i before admission for AMI, the study cohort was divided into two groups: SGLT2-i users (*n* = 111) and non-SGLT2-i users (*n* = 535). Baseline characteristics are shown in Table 1. The median age of the overall population was 70 years (range 61–79), while the SGLT2-i group was younger, with a median of 66 years (range 59–73). Over three-quarters of the patients enrolled were male, and more than 26% had a history of AMI. There was no difference in cardiovascular risk factors, history of established cardiovascular disease, or drug therapy between groups, yet the use of sulfonylureas was lower in the SGLT2-i user group. With respect to clinical data at admission and procedural data, more than half of patients experienced NSTEMI (equally distributed between the two groups), and there were no differences in terms of Killip class at presentation, history of AF, VT or cardiac arrest, radial vascular access, multivessel disease, or complete revascularization between groups. A summary of the clinical and procedural data is provided in Table 2. No differences were found between SGLT2-i users and non-SGLT2-i users in the occurrence of electrolyte imbalances; no differences in potassium, calcium, and magnesium concentrations that could potentially impact the onset of arrhythmias. Admission heart rate was significantly lower in the group of patients receiving SGLT2-i (Table 2). Although there were no differences in Hb1Ac levels on admission, median admission blood glucose levels were significantly higher in the non-SGLT2-i user group (185 vs. 158 mg/dL, *p* = 0.007). Furthermore, subcutaneous (s.c.) and intravenous (i.v.) insulin therapy was less frequently used during hospitalization in the SGLT2-i users (51.4 vs. 73.6%, *p* < 0.01; 15.3 vs. 26.9%, *p* = 0.010, respectively). Albeit there were marked differences in glycemic control and insulin use, no difference in terms of glycemic variability between the two groups was found. In addition, although there was a trend toward lower mean SD and coefficient of variation (CV) in the group of SGLT2-i users, these differences did not reach significance (SD 44.7 vs. 51.0, *p* = 0.20; CV 0.25 vs. 0.27, *p* = 0.23).

**Table 1:** Baseline characteristics of study population according to groups eligibility criteria.

	**Total**	**SGLT2-i users**	**Non-SGLT2-i users**	***P*** **-value**
	**(*N =* 646)**	**(*N =* 111)**	**(*N =* 535)**	
Age, median (IQR)	70 (61–79)	66 (59–73)	72 (62–80)	<0.001
Male Sex, *n* (%)	498 (77.1)	90 (81.1)	405 (75.7)	0.222
BMI	27.7 (25–31.3)	27.1 (24.6–30)	27.7 (25–31.4)	0.245
Smoking, *n* (%)	370 (57.3)	67 (60.4)	303 (56.6)	0.470
Hypertension, *n* (%)	541 (83.7)	98 (88.3)	443 (82.8)	0.154
Dyslipidemia, *n* (%)	508 (78.6)	90 (81.1)	418 (78.1)	0.490
PAD, *n* (%)	82 (12.7)	16 (14.4)	66 (12.3)	0.550
COPD, *n* (%)	90 (13.9)	15 (13.5)	75 (14)	0.889
CKD, *n* (%)	58 (9)	10 (9)	47 (8.8)	0.886
Previous TIA/CVA, *n* (%)	52 (8)	10 (9)	42 (7.9)	0.683
Previous AMI, *n* (%)	169 (26.2)	30 (27)	136 (25.4)	0.724
Previous PCI, *n* (%)	183 (28.3)	35 (31.5)	144 (26.9)	0.322
Antiplatelets, *n* (%)	321 (49.7)	60 (54.1)	261 (48.8)	0.312
Anticoagulation, *n* (%)	55 (8.5)	6 (5.4)	49 (9.2)	0.197
RAAS, *n* (%)	378 (58.5)	69 (62.2)	309 (57.8)	0.391
Diuretics, *n* (%)	196 (30.3)	31 (27.9)	165 (30.8)	0.543
B-blockers, *n* (%)	296 (45.8)	55 (49.5)	241 (45)	0.386
CCB, *n* (%)	197 (30.5)	35 (31.5)	162 (30.3)	0.794
Statins, *n* (%)	329 (50.9)	61 (55)	268 (50.1)	0.351
Low/moderate intensity	238 (72.3)	39 (63.9)	199 (74.3)	0.104
High intensity	91 (27.7)	22 (36.1)	69 (25.7)	
Ezetimibe, *n* (%)	78 (12.1)	15 (13.5)	63 (11.8)	0.609
Metformin, *n* (%)	467 (72.3)	80 (72.1)	387 (72.3)	0.955
Sulfonylureas, *n* (%)	166 (25.7)	13 (11.7)	153 (28.6)	0.001
DPP-4 Inhibitors, *n* (%)	54 (8.4)	8 (7.2)	46 (8.6)	0.630
GLP-1 Agonist, *n* (%)	19 (2.9)	5 (4.5)	14 (2.6)	0.284

Continuous variables are presented as mean ± SD or as median (IQR); categorical variables as number (%). AMI, acute myocardial infarction; BB, B-blockers; BMI, Body Mass Index; CCB, Calcium Channel Blockers; CKD, chronic kidney disease with 30 < GFR < 60 ml/min; COPD, chronic obstructive pulmonary disease; CVA, cerebrovascular accident; DPP-4, Dipeptidyl peptidase 4; GLP-1, Glucagon-like peptide-1; PAD, peripheral artery disease; PCI, Percutaneous Coronary Intervention; RAAS: renin-angiotensin-aldosterone system; TIA, transient ischaemic attack.

**Table 2:** Clinical admission, procedural and angiographic characteristics.

	**Total**	**SGLT2-i users**	**Non-SGLT2-i users**	***P*** **-value**
	**(*N =* 646)**	**(*N =* 111)**	**(*N =* 535)**	
STEMI, *n* (%)	309 (47.8)	52 (46.8)	257 (48)	0.819
ECG– balloon time (STEMI)	3 (2–5)	3 (2–6)	3 (2–5)	0.648
SBP	140 (125–160)	140 (125–155)	140 (125–160)	0.639
DBP	80 (70–90)	83 (70–90)	80 (70–90)	0.551
HR	81 (70–94)	75 (68–86)	83 (72–95)	<0.001
NYHA > 2, *n* (%)	113 (17.5)	16 (14.4)	101 (18.9)	0.266
Admission LVEF	47 ± 11	48 ± 10	47 ± 11	0.183
Killip Class ≥ 2, *n* (%)	135 (20.9)	18 (16.2)	117 (21.9)	0.183
VT/VF, *n* (%)	21 (3.3)	2 (1.8)	19 (3.6)	0.344
AF, *n* (%)	58 (9)	9 (8.1)	49 (9.2)	0.725
Admission blood glucose	180 (143–239)	158 (139–205)	185 (146–246)	0.007
K+, mmol/L (mean ± SD)	4 ± 0.3	4.1 ± 0.5	3.9 ± 0.2	0.342
Ca2+, mg/dl (mean ± SD)	9.6 ± 2.3	9.9 ± 2	9.4 ± 2.4	0.087
Mg, mg/dl (mean ± SD)	2 ± 0.3	2.1 ± 0.4	2 ± 0.2	0.854
Radial access, *n* (%)	542 (83.9)	92 (82.9)	450 (84.1)	0.748
LM lesion, *n* (%)	34 (5.3)	5 (4.5)	29 (5.4)	0.694
LAD lesion, *n* (%)	361 (55.9)	64 (57.7)	297 (55.5)	0.679
CX lesion, *n* (%)	168 (26)	33 (29.7)	135 (25.2)	0.326
RCA lesion, *n* (%)	203 (31.4)	35 (31.5)	168 (31.4)	0.979
1 Vessel lesion, *n* (%)	271 (42)	52 (46.8)	219 (40.9)	0.251
2 Vessels lesion, *n* (%)	231 (35.8)	33 (29.7)	198 (37)	0.145
3 Vessels lesion, *n* (%)	140 (21.7)	24 (21.6)	116 (21.7)	0.989
TIMI Flow pre, (mean ± SD)	1.2 ± 1.1	1.1 ± 1	1.2 ± 1.1	0.948
TIMI Flow post, (mean ± SD)	3 ± 0.3	3 ± 0.3	3 ± 0.3	0.678
Complete revascularization, *n* (%)	406 (62.8)	86 (77.5)	426 (79.6)	0.611

Continuous variables are presented as mean±SD or as median (IQR); categorical variables as number (%). AF, Atrial Fibrillation; CX, circumflex artery; DBP, diastolic blood pressure; ECG, electrocardiogram; HR, heart rate; NYHA, New York Heart Association; LAD, left anterior descending artery; LM, left main; LVEF, Left ventricular ejection fraction; PCI, Primary Percutaneous Coronary Intervention; RCA, right coronary artery; SBP, systolic blood pressure; STEMI, ST-elevation myocardial infarction; TIMI, Thrombolysis in Myocardial Infarction; VF, Ventricular Fibrillation; VT, Ventricular Tachycardia.

### In-hospital clinical outcomes

**Figure 1:**
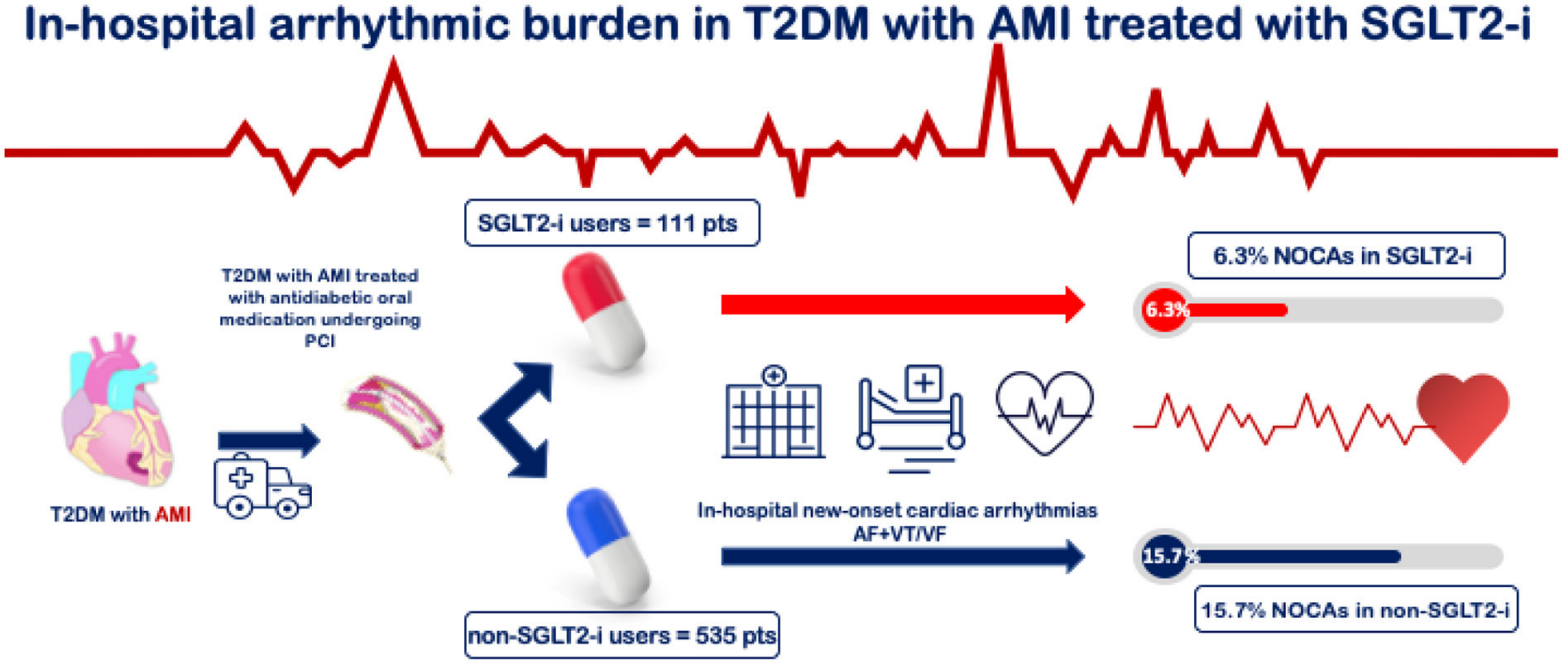
Study design and occurrence of new-onset cardiac arrhythmias. AF, atrial fibrillation; AMI, acute myocardial infarction; NOCAs, new-onset cardiac arrhythmias; T2DM, type 2 diabetes mellitus; SGLT2-i, Sodium-glucose co-transporter 2 inhibitors; VF, Ventricular Fibrillation; VT, Ventricular Tachycardia.

**Figure 2:**
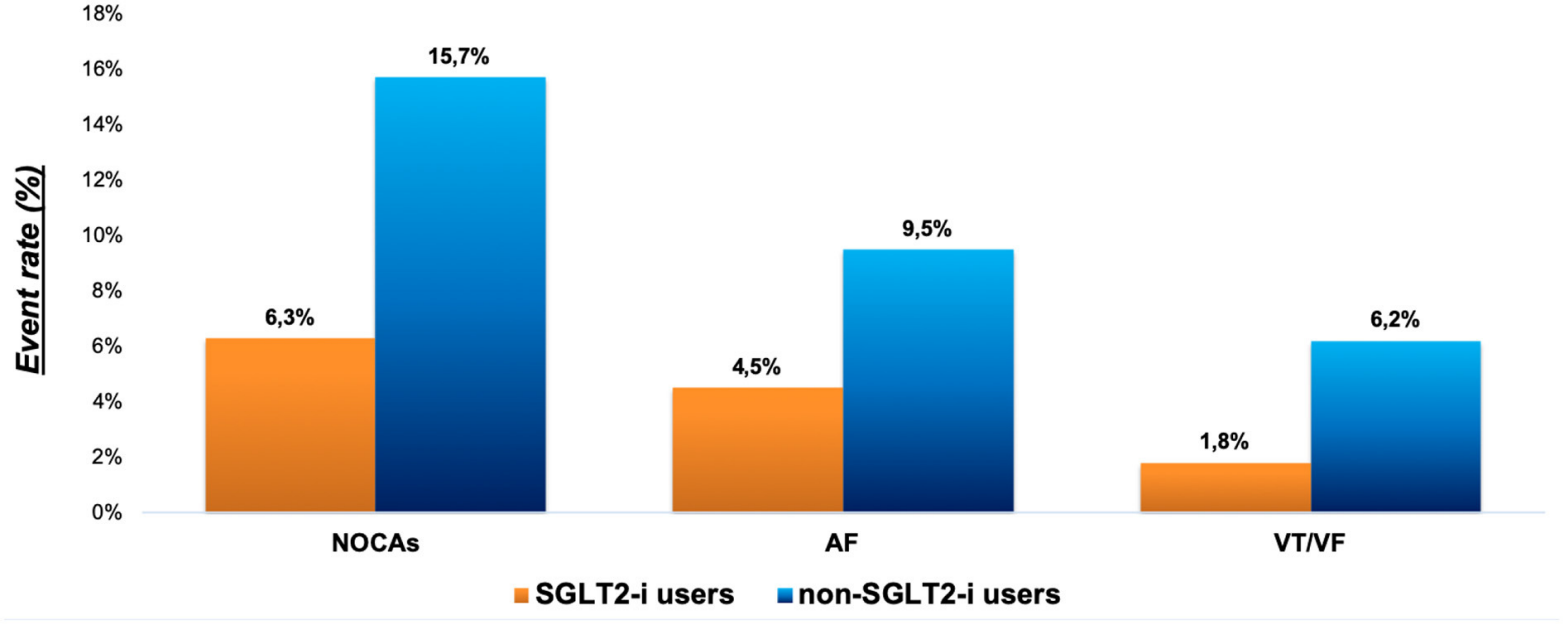
Primary endpoint and its individual components in SGLT2-i and non-SGLT2-i users. AF, atrial fibrillation; NOCAs, new-onset cardiac arrhythmias; SGLT2-i, Sodium-glucose co-transporter 2 inhibitors; VF, Ventricular Fibrillation; VT, Ventricular Tachycardia.

**Figure 3:**
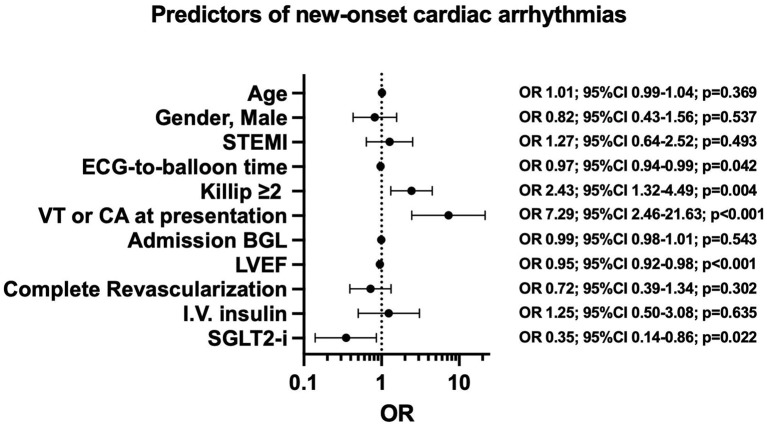
Effects of predictors of new-onset cardiac arrhythmias. BGL, blood glucose level; CA, cardiac arrest; ECG, electrocardiogram; LVEF, Left ventricular ejection fraction; NOCAs, new-onset cardiac arrhythmias; SGLT2-i, Sodium-glucose cotransporter 2 inhibitors; STEMI, ST-elevation myocardial infarction; VT, ventricular tachycardia.

The median length of stay was 5 days (range 4–8), with no difference between SGLT2-i users and non-users. A total of 91 (14.1%) NOCAs, 56 (8.7%) new-onset AF, and 35 (5.4%) new-onset VT/VF events occurred during hospitalization. SGLT2-i users had a lower rate of primary endpoint compared with non-SGLT2-i users (6.3 vs. 15.7%, *p* = 0.010) (Figure 1). Furthermore, a lower rate of AF and VT/VF was observed in the SGLT2-i users group compared with the non-users at Pearson Chi-Square test (4.5 vs. 9.5% and 1.8 vs. 6.2%, respectively; *p* = 0.032) (Figure 2). There were no significant differences in terms of recurrent AMI (*p* = 0.84) or the need for circulatory support (*p* = 0.98) between groups, whereas SGLT2-i users had less frequently post-procedure renal function impairment (*p* = 0.022) (Table 3). The hierarchical univariate analysis revealed an unadjusted reduction of 63% (OR = 0.37; 95%CI 0.17–0.83; *p* = 0.015) in the risk of the primary endpoint for SGLT2-i users compared with non-SGLT2-i users. In the multivariate logistic regression model, after adjusting for all known confounders, the use of SGLT2-i was independently associated with the lower occurrence of the composite endpoint of NOCAs (OR = 0.35; 95%CI 0.14–0.86; *p* = 0.022), together with no-VAs at presentation, reduced ECG-to-balloon time, higher LVEF, and Killip class <2 at admission (Figure 3). When each component of the composite endpoint was separately appraised by multinomial logistic regression, after adjusting for potential confounders, SGLT2-i therapy remained an independent predictor of lower VT/VF occurrence (OR = 0.20; 95%CI 0.04–0.97; *p* = 0.046) but not for AF occurrence (Table 4), which showed a reduction, without reaching statistical significance (OR = 0.40; 95% CI 0.14–1.14; *p* = 0.086).

**Table 3:** In-hospital outcomes of SGLT2-i users vs. non-SGLT2-i users.

	**Total**	**SGLT2-i users**	**Non-SGLT2-i users**	***P*** **-value**
	**(*N =* 646)**	**(*N =* 111)**	**(*N =* 535)**	
Hospital stay, days	5 (4–8)	5 (4–8)	5 (4–8)	0.526
hs-TnI max	2,368 (625–9,224)	903 (278–2,438)	3,155 (731–9,223)	<0.001
HbA1c	51 (45–59)	52 (48–57)	50 (44–60)	0.137
SD	49.8 ±46.5	44.7 ±50.2	51 ±45.6	0.206
CV	0.27 ±0.19	0.25 ±0.20	0.27 ±0.19	0.234
**In-hospital glucose-lowering strategy**				
Insulin s.c., *n* (%)	430 (66.6)	57 (51.4)	394 (73.6)	<0.001
Insulin i.v., *n* (%)	65 (10.1)	17 (15.3)	144 (26.9)	0.010
**In-hospital outcomes**				
Arrhythmia, *n* (%)	91 (14.1)	7 (6.3)	84 (15.7)	0.010
New-onset AF, *n* (%)	56 (8.7)	5 (4.5)	51 (9.5)	
VT/VF, *n* (%)	35 (5.4)	2 (1.8)	33 (6.2)	
Re-AMI, *n* (%)	7 (1.1)	1 (0.9)	6 (1.1)	0.838
Re-PCI, *n* (%)	13 (2.0)	4 (3.6)	9 (1.7)	0.190
IABP, *n* (%)	23 (3.6)	4 (3.6)	19 (3.6)	0.978
CI-AKI, *n* (%)	68 (10.5)	6 (5.4)	70 (13.1)	0.022

Continuous variables are presented as mean±SD or as median (IQR); categorical variables as number (%). AF, Atrial Fibrillation; AMI, Acute Myocardial Infarction, CI-AKI, Contrast-Induced Acute Kidney Injury; Hs-TnI, high sensitivity Troponin I; IABP, Intra-Aortic Balloon Pump; i.v., intravenous; MACE, major adverse cardiovascular events; PCI, Primary Percutaneous Coronary Intervention; s.c., subcutaneous; VF, Ventricular Fibrillation; VT, Ventricular Tachycardia.

**Table 4:** Multivariable analysis: Predictors of NOCAs, AF, VT/VF.

**NOCAs**			**AF**			**VT/VF**		
**Variables**	**OR (CI, 95%)**	***p*** **-value**	**Variables**	**OR (CI, 95%)**	***p*** **-value**	**Variables**	**OR (CI, 95%)**	***p*** **-value**
Age	1.01 (0.99–1.04)	0.369	Age	1.02 (0.98–1.05)	0.267	Age	0.99 (0.96–1.04)	0.972
Gender, male	0.82 (0.43–1.56)	0.537	RWMA	0.82 (0.24–1.90)	0.455	RWMA	0.76 (0.18–3.13)	0.702
STEMI	1.27 (0.64–2.52)	0.493	STEMI	1.83 (0.84–3.98)	0.124	STEMI	0.71 (0.23–2.27)	0.570
ECG-to-balloon time	0.97 (0.94–0.99)	0.042	ECG-to-balloon time	0.97 (0.93–1.00)	0.062	ECG-to-balloon time	0.96 (0.90–1.02)	0.194
Killip≥2	2.43 (1.32–4.49)	0.004	Killip≥2	2.34 (1.15–4.77)	0.019	Killip≥2	2.54 (0.95–6.85)	0.064
VT or CA at presentation	7.29 (2.46–21.63)	<0.001	VT or CA at presentation	2.77 (0.67–11.42)	0.160	VT or CA at presentation	17.3 (4.99–60.0)	<0.001
Admission BGL	0.99 (0.98–1.01)	0.543	Peak Hs-TnI, ng/L	1.00 (1.00–1.01)	0.776	Peak Hs-TnI, ng/L	1.00 (1.00–1.01)	0.267
LVEF	0.95 (0.92–0.98)	<0.001	LVEF	0.94 (0.90–0.97)	<0.001	LVEF	0.95 (0.91–0.99)	0.043
Complete revascularization	0.72 (0.39–1.34)	0.302	Complete revascularization	0.75 (0.38–1.48)	0.401	Complete revascularization	0.62 (0.24–1.57)	0.310
I.V. insulin	1.25 (0.50–3.08)	0.635	I.V. insulin	1.12 (0.56–2.21)	0.756	I.V. insulin	0.75 (0.29–1.91)	0.547
SGLT2-i	0.35 (0.14–0.86)	0.022	SGLT2-i	0.40 (0.14–1.14)	0.086	SGLT2-i	0.20 (0.04–0.97)	0.046

BGL, blood glucose level; CA, cardiac arrest; ECG, electrocardiogram; Hs-TnI, high sensitivity Troponin I; LVEF, Left ventricular ejection fraction; SGLT2-i, Sodium-glucose cotransporter 2 inhibitors; RWMA, regional wall motion abnormalities; STEMI, ST-elevation myocardial infarction; VT, ventricular tachycardia.

## Discussion

The main findings of our study can be summarized as follows: (i) in the context of diabetic patients with AMI, the new-onset of in-hospital major arrhythmic events, as the composite endpoint, was significantly lower in SGLT2-i users compared to non-SGLT2-i users; (ii) when considered separately, AF and VT/VF events occurred less frequently in the group of patients on SGLT2-i therapy compared with non-SGLT2-i users; (iii) SGLT2-i was a significant predictor of lower incidence of NOCAs, independently of age, sex, STEMI presentation, complete revascularization, admission stress hyperglycemia, and use of i.v. insulin therapy. Conversely, longer ECG-balloon time, Killip class >2, VT or cardiac arrest as presentation and lower LVEF turn out to be independent predictors of higher arrhythmic burden; (iv) after adjusting for all confounding factors, the use of SGLT2-i was identified as an independent predictor of reduced VT/VF occurrence. Our study is the first to report the impact of SGLT2-i therapy on arrhythmic burden in a specific setting of AMI diabetic patients.

The relationship between SGLT2-i therapy and cardiac arrhythmias has been extensively studied in T2DM patients with HF, but reported results have been inconsistent (22, 24). A systematic review and meta-analysis of randomized controlled trials on SGLT2i in T2DM patients highlighted that no significant difference was noted in the occurrence of AF in SGLT2-i patients (26). A later and larger meta-analysis (27) confirmed no benefit in protecting from new-onset AF in diabetic patients treated with SGLT2-i compared to other glucose-lowering drugs. These results were confirmed by two large real-world observational studies (28, 29), which found no significant differences in the occurrence of AF when SGTL2-i were compared with dipeptidylpeptidase-4 (DPP-4) inhibitors (30). By contrast, a larger body of evidence supports a protective effect in terms of new-onset AF (31–37). A post-hoc study from the Declare-TIMI 58 trial showed that dapagliflozin was associated with a lower incidence of new-onset AF in high-risk diabetic patients, with a relative risk reduction of 19% (31). Ling et al. (32) demonstrated a lower incidence of AF when SGLT2-i were compared to DPP-4-inhibitors (32). Several meta-analyses have confirmed that SGLT2-i could significantly decrease the incidence of AF both in diabetic and non-diabetic patients, with a reduction rate ranging from 19 to 25 % (33–36). These results appear to be consistent in recent observational studies (32, 37). Moreover, there seem to be no differences in protection from cardiac arrhythmias among different SGLT2-i, assuming a class effect (38). Less robust data regarding ventricular arrhythmias have been reported, possibly due to the large heterogeneity of the populations studied (22, 24). Overall, there are encouraging data on reducing VAs in patients treated with SGLT2-i (35, 39). In a post hoc analysis of DAPA-HF, dapagliflozin was shown to reduce the risk of any serious VAs or sudden cardiac death when added to standard therapy in patients with HF with reduced ejection fraction (HFrEF) (39). In this analysis, the relative risk reduction of VAs (as a composite of VT and VF) occurrence was 21%, and the effect was consistent across each component of the composite outcome. Other meta-analytic data highlighted a protective effect of SGLT2-i on the occurrence of VT, showing no differences across comorbidities or baseline conditions (35). There are also conflicting results on the incidence of VAs in patients receiving SGLT2-i; in fact, a recent meta-analysis found no association between SGLT2i therapy and lower VAs in patients with T2DM and/or HF and/or chronic kidney disease (34).

Although numerous—yet conflicting—the aforementioned data concern patients with or without T2DM, most with HF and HFrEF, in a chronic setting. Data on the acute effects of dapagliflozin in T2DM patients with HFrEF were provided by Ilyas et al. (40), who observed a reduced ventricular ectopic burden, suggesting an early anti-arrhythmic benefit after 2 weeks of treatment (40). Nevertheless, none of these relate specifically to patients with AMI. Our study is distinguishable from these previous reports inasmuch as we investigated the impact that chronic SGLT2-i therapy may have on patients experiencing hospitalization for AMI. In our cohort, in-hospital NOCAs were significantly reduced by 64%; in particular, a more effective reduction in VAs drove this finding. In fact, SGLT2-i seemed to reduce the risk of developing VAs by 80%, with a trend in reducing the new-onset of AF that did not reach significance. It is pertinent to emphasize how this finding is clinically relevant since VAs are frequently due to ischemia and the resulting molecular mechanisms during AMI, as they are closely related to sudden cardiac deaths in the early stages of AMI (5, 12). This observation is also significant considering that patients who develop any cardiac arrhythmia, especially VAs, during AMI have increased risks of subsequent arrhythmic events after discharge and a worse long-term prognosis (41). In terms of reduction in arrhythmic events, our results are in line with a nationwide population-based longitudinal Taiwanese cohort study that showed a reduction in total new-onset arrhythmic events, but not of all of its components, among SLGT2-i users (42). This study found no differences regarding AF, supraventricular arrhythmias, and VAs when considered separately (42).

We can relate this benefit in terms of NOCAs reduction to several mechanisms and pathways involved in the pathogenesis of cardiac arrhythmias on which SGLT2-i may impact. The EMBODY trial (43) investigated the effects of SGLT2-i on cardiac sympathetic and parasympathetic activities in patients with AMI and T2DM. Using several surrogate indices, the authors demonstrated that the empagliflozin group experienced significant improvement in both cardiac sympathetic and parasympathetic nerve activities, with a focus on sympathetic activity that might promote the development of arrhythmias (43). The sympathetic activity might be involved in another mechanism of control by SGLT2-i (44). In our cohort, the improved modulation of sympathetic tone could be reflected by a lower heart rate in patients treated with SGLT2-i. Indeed, previous reports have shown a more balanced autonomic system activity in patients treated with SGLT2-i when compared with non-SGLT2-i users, also in patients without structural heart disease (19). Another beneficial effect of SGLT2-i is protection against hyperglycemia-induced sympathetic overstimulation (45, 46). Different, non-mutually exclusive, mechanisms might mediate the relationship between insulin therapy and cardiac arrhythmias. First, hyperinsulinemia occurring during insulin therapy might induce sympathetic nervous system stimulation and consequently increase heart rate and QTc, which are well-known risk factors for ventricular arrhythmias (47–49). Additionally, patients treated with insulin are much more exposed to perceived and unperceived hypoglycemic crises (50). It should be pointed out that the presence of hypoglycemia is known to increase circulating levels of several counter-regulatory hormones (e.g., catecholamines), which increase the release of glucose from the liver and allow recovery from hypoglycemia. However, the same catecholamines also target the heart, resulting in increased heart rate and QTc, with a higher outcome consisting of a greater prevalence and incidence of cardiac arrhythmias (50). Accordingly, our patients treated with SGLT2-i exhibited lower admission blood glucose levels than patients treated with other OAD agents. Moreover, the lower number of hypoglycemic episodes associated with reduced insulin therapy (both subcutaneous and intravenous) resulting from minor stress admission hyperglycemia further corroborated the reduced in-hospital occurrence of arrhythmias in SGLT2-i users. Nevertheless, blood glucose levels at admission and the use of i.v. insulin did not appear to be predictor of arrhythmic events in our analysis. Glycemic control might be involved in the anti-arrhythmic action of SGLT2-i, but the hypothesis that these drugs also prevent arrhythmias *via* glucose-independent pathways is an added advantage since these drugs are also administered to non-diabetic patients. SGLT2-i ability to affect some of the ionic currents in cardiomyocytes could also explain some of their anti-arrhythmic effects. In particular, SGLT2-i could attenuate the increase in I_Na − late_ in diabetic patients with and without HF, thus preventing ventricular repolarization prolongation and early afterdepolarization (51). In ventricular cardiomyocytes isolated from rats, dapagliflozin attenuated the decreased I_K_ responsible for the potential prolongation and was thereby able to reduce the risk of cardiac arrhythmias in these preclinical models (52). SGLT2-i have also been shown to interact with Ca^2+^/calmodulin-dependent protein kinase II (CaMKII) (53), decreasing RyR2 phosphorylation thus reducing spontaneous diastolic Ca^2+^ release and sodium influx, both known to trigger delayed afterdepolarizations (54).

Finally, focusing on arrhythmias related to ischemia and reperfusion, it is relevant to highlight the results of an animal model that might reproduce our proposed acute ischemia setting (55). Hu et al. (55) aimed to investigate the impact of empagliflozin on myocardial ischemia/reperfusion-provoked arrhythmias *in vivo*. They found that pretreatment with empagliflozin protected from VAs induced by ischemia and reperfusion injury, and this benefit seems to be related to the activation of the ERK1/2-dependent signaling pathway (involved in cell survival processes) in a glucose-independent manner. Similarly, in ischemia-reperfusion rabbit models, empagliflozin was shown to reduce VAs, improving calcium cycling and mitochondrial fitness (56).

Several other molecular effects, as well as sodium hydrogen antiporter 1 (NHE1) receptor interaction, impact on oxidative stress modulation, myocardial extracellular matrix remodeling, and systemic inflammation, have been hypothesized to have a cardioprotective effect. In fact, we have actually demonstrated that AMI patients treated with SGLT2-i display a significantly lower inflammatory response and smaller infarct size than those receiving other OAD agents, independent of glucose control (25).

To date, real-world data on the cardioprotective effects and arrhythmia prevention of SGLT2i are mainly about diabetic patients, given that SGLT2-i prescription was only recently extended to non-diabetic patients. Therefore, further studies are needed to evaluate the effects of this class of drugs in the early stages of AMI, even in non-diabetic patients.

### Study limitations

The results of our study should be interpreted in light of some limitations. First, our conclusions are limited by the study's observational design, so our study results should be considered hypothesis-generating. Second, the sample size was powered to evaluate only a “class effect” but not the “doses effect”. However, the sample size estimation was made by considering long-term all-cause death as the main outcome. The present study is a sub-analysis evaluating arrhythmic events, so the sample size may be underpowered. Further investigation will be necessary to determine the finding that should be considered as hypothesis-generating. Third, categorization into SGLT2-i users and non-SGLT2-i users was based on the patients' reported therapy at admission and medical records. However, we cannot determine the effective adherence to treatment with SGLT2i or other OAD drugs. Moreover, the risk of potential concealed conditions must be taken into account. These may not have been included in the multivariable model. Finally, a unified protocol for cardiac arrhythmia detection was not observed in the medical records. Therefore, the possibility of undetected arrhythmia cannot be ruled out in this study.

## Conclusions

In a real-world clinical scenario of diabetic patients with AMI, the use of SGLT2-i was associated with a lower risk of new-onset cardiac arrhythmias during the in-hospital phase. In particular, the major effect seemed to be exerted on the reduction of VAs. Our findings are hypothesis-generating, and they turn on the spotlight on the pleiotropic and additional benefits of this class of drugs in patients with atherosclerotic cardiovascular disease, beyond their underlying role in controlling glycemic metabolism.

## Data availability statement

The raw data supporting the conclusions of this article will be made available by the authors, without undue reservation.

## Ethics statement

The studies involving human participants were reviewed and approved by Institutional Review Board of the IRCCS Azienda Ospedaliero-Universitaria of Bologna, Registration Number 600/2018/Oss/AOUBo. The patients/participants provided their written informed consent to participate in this study.

## Author contributions

AC and FG contributed conception and design of the study. PP, LB, EG, NMi, NMo, FG, MA, AS, SA, GE, and AI organized the database and collected data. AC and LB performed the statistical analysis. AC, FG, and PC wrote the first draft of the manuscript. PP and LB wrote sections of the manuscript. GS, CS, AF, GC, CM, EB, NMi, NMo, JO, DV, CP, PC, and RM revised the article and approved the final version of the manuscript. All authors contributed to manuscript revision, read and approved the submitted version.

## Funding

PP and GE report receiving a research grant from the CardioPaTh Ph.D. Program.

## Conflict of interest

The authors declare that the research was conducted in the absence of any commercial or financial relationships that could be construed as a potential conflict of interest.

## Publisher's note

All claims expressed in this article are solely those of the authors and do not necessarily represent those of their affiliated organizations, or those of the publisher, the editors and the reviewers. Any product that may be evaluated in this article, or claim that may be made by its manufacturer, is not guaranteed or endorsed by the publisher.
